# Satisfaction with pediatric telehealth according to the opinions of children and adolescents during the COVID-19 pandemic: A literature review

**DOI:** 10.3389/fpubh.2023.1145486

**Published:** 2023-04-06

**Authors:** Gergana Damianova Kodjebacheva, Taylor Culinski, Bushra Kawser, Saman Amin

**Affiliations:** ^1^College of Health Sciences, University of Michigan-Flint, Flint, MI, United States; ^2^Institute for Healthcare Policy and Innovation, University of Michigan-Ann Arbor, Ann Arbor, MI, United States; ^3^Department of Behavioral Sciences, College of Arts and Sciences, University of Michigan-Flint, Flint, MI, United States

**Keywords:** telehealth, telemedicine, adolescents, teenagers, children, attitudes, perceptions, satisfaction

## Abstract

**Objective:**

To review satisfaction with telehealth among children and adolescents based on their own opinions during the COVID-19 pandemic.

**Methods:**

In the PubMed, CINAHL, PsycINFO, and Embase databases, we searched for peer-reviewed studies in English on satisfaction with telehealth among children and adolescents (rather than parents). Both observational studies and interventions were eligible. The review was categorized as a mini review because it focused on the limited time frame of the COVID-19 pandemic. We followed the Preferred Reporting Items for Systematic Reviews and Meta-Analyses guidelines. Reviewers extracted information from each study and assessed risk of bias.

**Results:**

A total of 14 studies were eligible. Studies were conducted in Australia, Canada, Italy, Israel, Poland, South Korea, the United Kingdom, and the United States. They focused on a variety of health conditions. Two of the 14 studies were interventions. Participants expressed high satisfaction with video and telephone visits and home telemonitoring while also preferring a combination of in-person visits and telehealth services. Factors associated with higher satisfaction with telehealth included greater distance from the medical center, older age, and lower anxiety when using telehealth. In qualitative studies, preferred telehealth features among participants included: a stable Internet connection and anonymity and privacy during telehealth visits.

**Conclusion:**

Telehealth services received favorable satisfaction ratings by children and adolescents. Randomized-controlled trials on the effectiveness of pediatric telehealth services compared to non-telehealth services may assess improvements in satisfaction and health outcomes.

## 1. Introduction

The use of telehealth increased during the COVID-19 pandemic (1). One telehealth definition is “the delivery of healthcare services, where distance is a critical factor, by all healthcare professionals using information and communication technologies for the exchange of valid information for diagnosis, and treatment and prevention of disease” (2). Synchronous telehealth care involves a live visit with a medical provider using video conferencing or a telephone call (3). Asynchronous telehealth includes the use of email, text messaging, and patient portals for the exchange of information (3). Remote monitoring involves receiving vitals and photographs from a patient for a certain health condition thus allowing for diagnosis and treatment (3, 4).

In pediatric care, telehealth was delivered through video and/or telephone consultations (5). It involved remote patient monitoring such as performing a bilateral smartphone otoscopy for the child at home (6) and measuring blood glucose levels at home and reporting the readings on a mobile application (7). Present-day Internet bandwidth (i.e., Internet speed/capacity) allows for video visits and remote monitoring without technological issues. Limited access to high-speed Internet for rural residents and those of lower socio-economic status, however, may increase barriers to quality remote healthcare (8).

Prior research focused on satisfaction with telehealth among adult patients and/or parents. In these studies of adult patients or parents, participants indicated technological issues such as the lack of a stable/fast Internet connection (9), lack of in-person interaction with the medical provider (10, 11), and lower empathy in care (11) as areas of concern. Parents who received care through in-person visits were more satisfied in the area of emotional support compared to those who received remote consultations (*p* = 0.039) (12).

In surveys, adolescents viewed themselves as technologically savvy and expressed a preference for technologically-enhanced interaction and learning (13). Telehealth may offer young people opportunities for enhanced medical experiences through telehealth. Understanding satisfaction with telehealth among young people (i.e., children and adolescents) can help develop healthcare improvements that meet the needs of youth.

To the best of our knowledge, no systematic review assessed satisfaction with telehealth among children and/or adolescents who offered their own opinions during the COVID-19 pandemic. A systematic review on randomized-controlled trials (RCTs) discovered high satisfaction with telehealth among parents and patients combined prior to the COVID-19 pandemic (14).

Our study conducted a systematic literature review on the satisfaction with telehealth among young people during the COVID-19 pandemic. It included studies where children and/or adolescents, rather than parents, answered questions on their satisfaction with telehealth. We classified the study as a mini review because it focused on a defined period of time (the COVID-19 pandemic), sought to provide succinct findings, offered recommendations for medical providers and healthcare administrators on preferred features of telehealth according to the suggestions by adolescents, and provided suggestions for future research.

## 2. Methods

### 2.1. Research question, review design, and eligibility criteria

The review question was: What is the satisfaction of young people (i.e., children and adolescents) with telehealth? We sought to follow the Preferred Reporting Items for Systematic Reviews and Meta-Analyses (PRISMA) 2020 (15) for this registered review (16). The PI (E) COS structure was:

- Outcome: Satisfaction.
- Participants: Pediatric participants who could answer items on their own, i.e., primarily adolescents. Younger children who answered questions on their own were also included. Studies that mixed children/adolescents and young adults in one category were included.
- Intervention/Exposure: Telehealth.
- Comparison group: Not required.
- Time frame: COVID-19 pandemic.

#### 2.1.1. Language

The inclusion criteria were peer-reviewed studies with full-text in English.

#### 2.1.2. Study designs

Due to the limited number of studies, we did not place restrictions on study design. The PRISMA guidelines describe: “PRISMA primarily focuses on the reporting of reviews evaluating the effects of interventions, but can also be used as a basis for reporting systematic reviews with objectives other than evaluating interventions” (15). All intervention designs were included. Observational cross-sectional, cohort, and case–control studies involving surveys and interviews were included. If the study did not specifically list the type of design used, decisions were made on the type of design based on descriptions in the article.

#### 2.1.3. Health conditions

Participants could be receiving services for and/or have any physical or mental health condition in this review.

#### 2.1.4. Time frame

Given that search words for the COVID-19 pandemic were utilized, the search was not restricted by date. The search was performed on December 15, 2022. Any studies, therefore, meeting the inclusion published by December 15, 2022 were included. The study had to specify that it focused on the period of the COVID-19 pandemic to be included.

#### 2.1.5. Participants

Keywords related to the participants in the search in the databased included pediatrics, pediatric, child, adolescent, and teen.

#### 2.1.6. Intervention/Exposure

No restriction on the type of telehealth was placed. Synonyms for the exposure in the search in the databases included: telehealth, telemedicine, remote consultation, and video consultation.

#### 2.1.7. Intervention/Exposure comparator

There was no requirement that the study should have a comparison group for in-person or other medical services.

#### 2.1.8. Outcome

Synonyms of the outcome in the search included satisfaction, attitudes, and perceptions. Studies that developed their own measurements and those with reliable and/or valid and/or other measurements on satisfaction were included.

#### 2.1.9. Databases, search strategy, synonyms of keywords

The literature review was performed in the PubMed, CINAHL (housed in EBSCO), EMBASE, and PsycINFO (housed in EBSCO) databases. It is recommended that to ensure search strings are reproducible, researchers collaborate with librarians (17). The search strings were developed with the guidance of a librarian from the University of Michigan library whose name was included in the Acknowledgments. In each database, the sets of words in Supplementary file S1 were used. Several of the search words (i.e., patient satisfaction, pediatrics, child, adolescent, telemedicine, remote consultation, and COVID-19) were entered as MeSH words in the PubMed database meaning that the database included its own associated synonyms. For example, the word telemedicine has the following synonyms as a MeSH term: telehealth, mobile health, tele-ICU, tele-intensive care, tele-referral, virtual medicine, eHealth, and mHealth.

### 2.2. Data extraction and synthesis

All resulting articles were uploaded into EndNote where automated removal of duplicates took place. The authors removed any remaining duplicates in Excel. Two authors (GDK and TC) reviewed all remaining titles and abstracts for inclusion in the review. The two reviewers resolved disagreements on inclusion after discussing the full-text of the article.

Characteristics of each included study were extracted in Tables 1, 2. Results that represented the overall study findings were extracted. BK and SA performed the data extraction in tables. GDK reviewed the data extraction. BK, SA, and GDK then all reviewed the extracted data and resolved disagreements through consensus.

**Table 1:** Location, number, age, and condition among participants in the 14 included studies on satisfaction with telehealth among children and adolescents based on their own opinions during the COVID-19 pandemic.

Study	Country	Number of young patients	Mean age or other information on age of participants	Health condition
Allison et al., 2022 (18)	United States	48 (Surveys) 14 (Interviews)	15	No specific health condition (primary care)
Bassi et al., 2022 (19)	Italy	152	17.9	Type 1 diabetes
Capri et al., 2021 (20)	Italy	11	5.82 (age range: 5–10 years)	Neuromuscular disorder; no children had cognitive impairments
Carroll et al., 2022 (21)	Australia	52	Median age: 14	Neuromuscular disorder
Choi et al., 2022 (22)	South Korea	13	13.8	Spina bifida
Hawke et al., 2021 (23)	Canada	409	15 to 28	Mental health and substance use (participants from both clinical and non-clinical samples were invited)
Mekori-Domachevsky et al., 2021 (24)	Israel	44	14.62 (SD: ± 2.12 years)	Mental health
Mikesell et al., 2022 (25)	United States	14	13 to 17	No specific health condition (primary care)
Randall et al., 2022 (26)	Australia	140	21	Schizophrenia, spectrum disorders and undifferentiated psychosis
Spigel et al., 2021 (27)	United States	73	19.1	Eating disorder
Stewart et al., 2021 (28)	United Kingdom	53	Youth <18	Eating disorder
Troncone et al., (2022) (29)	Italy	Total: 610 patients Video visit: 305 In-person: 305	12.17	Type 1 Diabetes
Wasilewska et al., 2022 (30)	Poland	21	12.8	Duchenne muscular dystrophy (DMD)
Ziani et al., 2022 (31)	Canada	19	Range: 14–25	No specific health condition (medicine, physiotherapy, speech therapy, and nutrition)

**Table 2:** Telehealth approach, assessment of satisfaction, and findings in the 14 included studies on satisfaction with telehealth among children and adolescents based on their own opinions during the COVID-19 pandemic.

Study and study design	Telehealth approach	Assessment of satisfaction: Description of data collection tools and reliability and validity	Findings
Allison et al., 2022 (18) Observational mixed-methods study: prospective and qualitative study	Video visits (09/2020–01/2021)	Description: Adolescents completed the Telemedicine Satisfaction and Usefulness Questionnaire and the Working Alliance Inventory (WAI) short version. Validity and reliability: The survey portion of this study utilized established measures. The issue on reliability/validity was not applicable for the second part of the study since qualitative interviews were used.	77% of adolescents (37) indicated that their last video visit was very private, 10% (21) somewhat private, and 2% (1) not private. The following are quotes from the interviews on technological difficulties: “I have had technological like issues in the past with my camera not working…,” and autonomy and independence: “And maybe because like I did it without my parents, so it was almost like, I was an adult.”
Bassi et al., 2022 (19) Observational prospective study	Video calls for follow-ups and education. Telenursing support on infusion pumps. Downloading information from insulin pumps and sharing health data with provider by the participant (01/2021–09/2021)	*Description:* Evaluation of satisfaction included statements such as: “I was able to explain my medical problems well enough *via* televisit.” Scores were determined on a scale of 0–10: Neutral (0–6) and High (>6). *Validity and reliability*: The questionnaire was developed by 10 healthcare professionals in the Endocrinology and Diabetes Centers of Giannina Gaslini Institute and inspired by a published survey.	10% of patients had a score of 0–6 on the item “I was able to explain my medical problems well enough *via* televisit” and 90% had a score of 7–10. 32% of patients had a score of 0–6 on the item “I think that televisits are an adequate modality of assistance for my disease” and 68.1% had a score of 7–10. Those who lived farther away from center expressed higher satisfaction with telehealth compared to those who lived closer (*p* = 0.017). High satisfaction with telehealth decreased when patients were asked whether video calls acted as a sufficient type of diabetes care.
Capri et al., 2021 (20) Quasi experimental one group post-test only study	The program included 12 web meetings (3 times per week over 1 month) consisting of games to enhance cognitive functions (March 11, 2020 –December 16, 2020)	*Description:* A total of 6 questions with close-ended responses on a 7-point Likert scale (from “Not as good” to “Very good”) such as: How happy were you to participate in the program?; How much did you like to play with the psychologists? *Validity and reliability*: Surveys were distributed *via* REDCap (an online survey tool provided by the Murdoch Children’s research institute). The survey questions were developed by neuromuscular clinic clinicians and members of the research team.	The median for “How happy were you to participate in the program?” was 7. The median for “How much did you like to play with the psychologists?” was 6.55.
Carroll et al., 2022 (21) Observational retrospective study	Healthdirect Australia video call system and telephone calls for follow-up appointments with a clinician (Study period: Not specified, COVID-19 emergency)	*Description:* Surveys consisted of multiple choice, sliding scale, and open-ended items. Patients used the Wong Baker faces ordinal scale as a rating system which ranged from 1 (happiest face) to 7 (saddest face). Patients were asked about their preferred future model of care. Patients answered items on topics of interaction/engagement with providers and differences between in person and telehealth visits. *Validity and reliability*: Questions were developed by using ideas from reliable published surveys.	58.8% of patients showed a preference of a hybrid model of telehealth which included both face-to-face neuromuscular clinic and telehealth appointments. 62.3% of patients reported feeling happy with telehealth appointment types. Patients found that it was easier to talk with the healthcare professional in person: 82.4% rated happy for the in-person appointments in comparison to the 43.1% who rated happy for the telehealth appointments (Wong Baker faces ordinal scale).
Choi et al., 2022 (22) Quasi experimental pre-test post-test study	Six video call sessions in an eHealth care nurse-led transition program (07/2021–09/2021)	*Description:* Program satisfaction was measured through a 10-item questionnaire. Survey for satisfaction was divided into 2 domains: (1) Content: lecture and group activity, mentorship; and (2) Methods: delivery, duration, and period. Satisfaction of each specific individual theme was rated through a 6-point scale: 1 (not helpful at all) and 6 (very helpful). Overall satisfaction was measured on a 10-point scale: 1 (strongly dissatisfied) and 10 (strongly satisfied). Participants of the survey ‘Satisfaction with the transition care program’ were able to leave comments and suggestions to questions such as, “What was particularly good or impressive about this program?” *Validity and reliability*: Multiple questionnaires based on concepts from the Life Course Model (containing 3 domains) were used. Validity and reliability of the satisfaction survey were not discussed.	The individual categories of lecture, group activity, and mentorship were rated from 4.9–5.3 indicating high level of satisfaction. Overall satisfaction was 9.1, indicating a high level of satisfaction with the eHealth program. Critiques of the program were the long sessions and difficult content/terms.
Hawke et al., 2021 (23) Observational qualitative study	Mental health and substance use virtual services (August 2020 wave)	*Description:* Participants answered close-ended questions on what type of technology they used. Then, they answered open-ended questions on the types of virtual services they preferred, what the preferred features of the virtual services were, and what the pros and cons of virtual services were. *Validity and reliability*: The research team along with consultants from local service providers developed the questions by using a conceptual framework. Young persons were consulted in the development of the survey. Most questions were qualitative and the issue of reliability/validity is not applicable.	Participants preferred these types of visits in order of preferred from highest to least preferred: video, phone or voice, text or chat support. Preferred technological features included: smooth video audio, stable Internet connection, ability to mute and turn on/off camera, and ability to share files. Participants expressed appreciation for anonymity and privacy. Participants expressed interest in complimentary, constant, and convenient methods for booking and availability of virtual services.
Mekori-Domachevsky et al., 2021 (24) Observational prospective study	Telepsychiatry through video conferencing format (03/2020–04/2020)	*Description:* Patients completed the Telemedicine Satisfaction Questionnaire (TSQ). *Validity and reliability:* Authors used multiple valid and reliable surveys, i.e., the Telemedicine Satisfaction Questionnaire (TSQ) (Cronbach’s alpha = 0.88).	Participants had a total score of 54.50 ± 19.50 on the TSQ, in which the highest subscore medians were in the areas of quality of care and perception.
Mikesell et al., 2022 (25) Observational qualitative study	Video appointments (11/2020–03/2021)	*Description:* Interviews with open-ended questions focused on level of satisfaction with teleconsultation as well as the perceived pros and cons of teleconsultation. *Validity and reliability:* The issue on reliability/validity was not applicable since qualitative interviews were used.	The following are some of the statements by the participants on: increased patient-centeredness: “I guess being at home makes me feel more secure and listened to…” and “I feel like they actually have more time to talk to you”; preference for in-person visits: “I would have preferred an in-person visit but I did feel comfortable with the [video] platform”; challenges with engagement with the physician: “I kind of felt like, it was like you answer the question and you move on to the next question, versus like it being a conversation.”
Randall et al., 2022 (26) Observational retrospective study	Telepsychiatry through video chat format and telephone interactions for multidisciplinary services by clinicians. (01/2020–05/2020)	*Description:* One question asked about preferences for mode of appointment (telepsychiatry only, face-to-face only, a combination, or undecided), and other six questions asked about experience of telepsychiatry (attitude, accessibility, clinical engagement, technology use and privacy/confidentiality), rated on a 5-point Likert scale (1 = strongly disagree to 5 = strongly agree) *Validity and reliability*: Validity and reliability of the surveys were not discussed.	Participants had mixed views about telepsychiatry. 42.2% (30) indicated preference for in-person appointments. 19.7% (14) preferred only telepsychiatry appointments. 36.6% (26) preferred a combination of face-to-face and telepsychiatry. 1 participant remained undecided. A preference for in-person appointments was linked to: younger age and anxiety when using telepsychiatry.
Spigel et al., 2021 (27) Observational prospective study	Video conferencing for eating disorder care and therapy (2020)	*Description:* The study sent web-based surveys through Research Electronic Data Capture (a HIPAA compliant database). Questions such as the following were used: “I have been able to access my providers;” Participants could then check all that applied: “*via* telehealth,” “in person,” “neither (“I have not been able to access my providers at all”). Responses were then dichotomized to demonstrate any access to care (*via* telehealth or in-person) vs. no access. *Validity and reliability*: Survey questions were developed by the authors.	In 64 patients who had telehealth access, 9% stated care was superior than usual, 59% stated care was as good as usual, 30% stated care was worse than usual, and 2% stated care was much worse. No association existed between access to care *via* telehealth and perceived disruption of care (*p* = 0.99).
Stewart et al., 2021 (28) Observational mixed-methods study: prospective and qualitative study	Video visit or phone calls (05/2020–07/2020)	*Description:* Likert items aided in investigating treatment experience such as how understood patients felt, degree to which extensive problems were addressed, the influence of technology, and efficacy of online interactions: 1 poor/not at all – 7 definitely. Participants also answered questions on their preferences for treatment once restrictions decreased (online, in-person, or dependent on needs). Four open-ended questions asked about pros and cons of online therapy and recommendations for enhancements. *Validity and reliability*: The satisfaction surveys were developed by the authors.	The median ratings on a 7-point scale post lockdown were: overall experience: 6, feel understood: 6, address important issues: 6, impact of technology: 4, benefited from online: 7. In qualitative responses, participants expressed gratitude for the relationships they developed with the medical providers. However, participants also expressed missing seeing their therapist in person and that something was missing from the process while using telehealth services.
Troncone et al., (2022) (29) Observational prospective study	Video visits for caregivers and patients (04/2020–05/2020) compared to in-person visit for caregivers and patients (06/2020 and 07/2020)	*Description:* Patients completed the Jefferson scale of patient perceptions of physician empathy (JSPPPE). *Validity and reliability*: Authors used a reliable and valid survey, i.e., the Jefferson scale of patient perceptions of physician empathy (Cronbach’s alpha = 0.896).	The mean JSPPPE score was 28.92 for video consultations and 27.82 for the in-person visits thus indicating mildly higher satisfaction with the video visit (p = 0.096).
Wasilewska et al., 2022 (30) Observational prospective study	Telemedicine monitoring. Telemonitoring of pulmonary function was performed by AioCare system. (06/2021–10/2021)	*Description:* The patients expressed their satisfaction with the AioCare home spirometry by using a 5-point scale, where “1” meant the worst and “5” meant the best score. Other questions focused on benefits and problems with measurements using home spirometry. *Validity and reliability*: Reliability and validity of satisfaction surveys were not presented.	The mean satisfaction with telemonitoring was 4.46/5 (SD 0.66) after 1 month and 4.91/5 (SD 0.28) after 3 months. The most reported benefit of home monitoring included that 38% had improvement in breathing after one month and 52% after 3 months of telemonitoring.
Ziani et al., 2022 (31) Observational qualitative study	Telephone or video appointments (08/2020 to 01/2021)	*Description:* Interviews with open-ended questions focused on level of satisfaction with teleconsultation as well as the perceived pros and cons of teleconsultation. *Validity and reliability*: The issue on reliability/validity was not applicable since qualitative interviews were used.	The participants had a preference for videoconferencing over telephone appointments. Among those who already had a developed relationship with a health professional, teleconsultation was perceived as an element of stability. Teleconsultation was perceived as a chance to avoid the stressful environment of healthcare entities. The study showed quotes from adolescents on their experiences with telehealth by age. The following were comments by patients younger than 18 years: “During the lockdown, my physician called me even if we did not have a scheduled appointment, he called me to know how I was doing, how I felt. So, I found it very comforting to know there was someone who wanted to know how I was despite all that was going on.” “The advantage is I was not at the hospital. The hospital was more stressful. We go there, we spend time in the waiting room, we wait, we go into the room, we do the things and after we leave. It was relaxing to be at home and in from of the screen instead of being in a hospital.”

### 2.3. Quality of evidence/risk of bias

The JBI critical appraisal tools (32) aided in assessing the quality of evidence in studies.

To assess particular limitations of studies, the review involved answering questions across study type based on JBI forms (32). Reviewers used separate forms to answer the following JBI questions:

- Were the criteria for inclusion in the sample clearly defined?
- Were the socio-demographic characteristics of participants described in detail?
- Was the time period (such as months and years) for telehealth services clearly defined?
- Were satisfaction measures valid and reliable?
- Were appropriate statistical/qualitative analyses used?

BK and SA first separately assigned risk of bias scores. GDK reviewed the scores, and BK, SA, and GDK resolved disagreements through consensus. A score of 1 indicated Yes, a score of 0.5 indicated Partially, and a score of 0 meant No or Unclear on the JBI form questions. Higher total scores meant lower risk of bias and lower total scores meant elevated risk of bias.

## 3. Results

### 3.1. Literature search

The Supplementary file S2 Figure illustrates the flow of selecting articles. The number of final studies included in this review was 14 (18–31). An example of a study that was excluded during the full-text review was the study of Elbin et al., 2022 (33) on the satisfaction with telehealth among adolescents prior to the pandemic. Another example of an excluded study during the full-text review included the study of Graziano et al., 2021 (34) where satisfaction of participants was presented together without differentiating between satisfaction between youth and parents.

### 3.2. Study characteristics

Studies were conducted in Italy (19, 20, 29), the United States. (18, 25, 27), Australia (21, 26), and Canada (23, 31) (Table 1). In addition, one study was conducted in each of these nations: South Korea (22), Israel (24), the United Kingdom (28), and Poland (30). Studies focused on a variety of health conditions such as general medicine, eating disorder, type 1 diabetes, mental health, and speech impairment (Table 1).

### 3.3. Quality and risk of bias assessment

#### 3.3.1. Study design

Among the 14 studies, there were 2 interventions (20, 22) (Table 2). Five were prospective cohort investigations where investigators classified individuals as participating in telehealth services and then followed them over time to assess their satisfaction (19, 24, 27, 29, 30). Two studies used retrospective analyses where answers to satisfaction surveys among individuals participating in telehealth at certain time periods in the past were assessed (21, 26). Three studies were observational qualitative (23, 25, 31). Two observational mixed-methods studies utilized both satisfaction surveys and qualitative interviews (18, 28).

#### 3.3.2. Outcome variable (i.e., satisfaction) reliability/validity

The authors used reliable and valid surveys (Table 2), such as the Jefferson scale of patient perceptions of physician empathy (Cronbach’s alpha = 0.896) (29). The Telemedicine Satisfaction Questionnaire (TSQ) (Cronbach’s alpha = 0.88) and the Working Alliance Inventory (WAI) (Cronbach’s alpha = 0.92 for the long version; Cronbach’s alpha = 0.89 for the WAI short version) were utilized (18, 24). Other authors developed the surveys themselves with or without the collaboration of consultants specializing in telehealth (19, 20, 22, 23, 27, 28).

#### 3.3.3. Quality of evidence scores based on JBI critical assessment tool common questions

Total scores among the 14 studies were distributed as follows: three had the highest quality of evidence score of 5 out of 5, two had a score of 4.5, five had a score of 4, three had a score of 3.5, and one had a score of 3 (Supplementary Table S2). Reasons for decreased scores included: limited description of the socio-demographic characteristics of the subjects and lack of valid and reliable measures of satisfaction.

### 3.4. Satisfaction with telehealth

#### 3.4.1. High satisfaction overall and factors associated with higher satisfaction

Participants tended to express high satisfaction with telehealth. For example, 85% of participants were able to relay their medical problems comfortably and felt that they received appropriate attention from the healthcare professionals during video visits in the study of Bassi et al. (19). In one study, for participants, who already had a relationship with a health professional, teleconsultation was perceived as a chance to circumvent the more stressful setting of healthcare entities (31). In the study of Troncone et al. (29), the mean Jefferson Scale of Perceptions of Physician Empathy (JSPPPE) score was 28.92 for video consultations and 27.82 for the in-person visits demonstrating slightly higher satisfaction with the video visit which was not statistically significant (*p* = 0.096) (30). Factors associated with higher satisfaction with telehealth included: longer distance from the health center (19), older age (26), and lower anxiety when using telehealth (26).

#### 3.4.2. Advantages of telehealth

Advantages of telehealth according to the participants included being comforting and less stressful, according to the findings of a qualitative study (31). Participants noted an increase in person-centeredness, specifically in their providers’ attentiveness and eye contact during their visit (25). The following were positive statements by adolescent patients on increased patient-centeredness through video visits: “I guess being at home makes me feel more secure and listened to” and “I feel like they actually have more time to talk to you” (25).

#### 3.4.3. Preferred telehealth features

In order of highest to least preferred, participants favored: video, phone, or voice, text, or chat support (23). Preferred technological features among participants included: stable Internet connection, ability to mute and turn on/off camera, and ability to share files. Participants expressed appreciation for anonymity and privacy. They preferred convenient methods for booking and increased availability of virtual services (23). Some participants wanted a combination of in-person and telehealth care. Specifically, 58.8% of participants demonstrated a preference of a hybrid model of telehealth which included both in-person and telehealth appointments (21).

#### 3.4.4. Surveying of very young patients

Investigators were able to survey participants as young as 5 (age range 5 to 10 years) by asking simple questions that included the use of a scale of happy faces. The median for “How much did you like to play with the psychologists?” was 6.55 (out of 7) (20).

#### 3.4.5. Challenges with telehealth

In some studies, participants expressed that it was easier to communicate with the medical provider during in-person compared to telehealth appointments because of being able to develop better rapport with the physician when in the same room. In the study of Carroll et al., 2021, using the Wong Baker faces ordinal scale, 82.4% rated happy for the in-person appointments in comparison to the 43.1% who rated happy for the telehealth appointments (21). In another study, 42.2% of participants indicated a preference for in-person appointments (26). Using qualitative responses, participants expressed missing seeing their therapist in person (28). In yet another qualitative investigation, the following were statements by adolescent patients on the preference for in-person visits: “I would have preferred an in-person visit but I did feel comfortable with the [video] platform” and the challenges with engaging the physician during video visits: “… It was like you answer the question and you move on to the next question, versus like it being a conversation” (25). Another quote by a young patient relating to the preference for in-person visits was: “… I prefer face-to-face because I find that it is always more concrete personally. And I think it’s always easier when it’s in person. … I think there’s a human side to it too because I think at the moment we are a bit lacking because of the COVID” (31).

Technological problems also made telehealth appointments challenging. Participants stated: “It’s different, of course, the sound quality. … Because you do not hear as well as on Zoom as you do in an in-person session. So I find it a disadvantage” (31) and “I have had technological like issues in the past with my camera not working ….” (18).

## 4. Discussion

### 4.1. Primary findings

The reviewed studies in this literature review presented the satisfaction of children and/or adolescents separately/distinctly from parents. Studies primarily included adolescents (either exclusively or combined with young adults) but one included patients as young as 5 years (20). They focused on a variety of pediatric care needs and specialties. Studies usually defined telehealth as video/telephone consultations/educational sessions or home telemonitoring. Two of the 14 studies were interventions (20, 22). In the current review, a trend of overall high satisfaction with telehealth existed although participants also expressed a preference for a mixture of in-person and virtual visits. Prior literature reviews did not explicitly focus on satisfaction with telehealth among children and/or adolescents (35–39).

In most studies in this review, participants answered questions on their experience with telehealth without comparing it to their experience with in-person visits. In one study, the authors did compare telehealth to in-person visits and discovered higher satisfaction with video visits compared to in-person visits among adolescents which was not statistically significant (27). The trend for superior satisfaction with telehealth encounters compared to in-person appointments was similar to the results of a pre-pandemic RCT (33). In the pre-pandemic RCT, 93% of adolescents rated the telehealth visit as enjoyable compared to 86% of adolescents in the in-person visit (33). The pandemic study (29) included in this review and the pre-pandemic study (33) did not assess potential reasons for adolescents’ preferences.

### 4.2. Limitations of the reviewed studies

A non-randomized design, small sample size, limited generalizability, convenience sampling, sampling from both clinical and non-clinical populations without presenting separate findings for the two, limited information on socio-demographic characteristics among participants, and lack of reliable and valid satisfaction assessment tools were limitations. Another limitation was that because the number of adolescents who could answer questions on their own was small, results for young adults aged 18–28 and adolescents were sometimes mixed. Most studies examined satisfaction with video conferencing.

### 4.3. Limitations of this review

Only peer-reviewed articles written in English were included. Additional manuscripts may have been published after the search date. Articles housed only in other databases would not be included in this review. Although MeSH terms (library terms that also search for main synonyms) were used as part of the search, additional synonyms of keywords may have resulted in more eligible studies. The results may not be applicable to developing countries. Yet another limitation is that the different study designs and measurements did not allow for the conduct of a meta-analysis.

### 4.4. Implications for practice

Participants preferred in-person appointments in some of the studies. In prior research, parents believed that a physical examination resulted in enhanced treatment adherence (12). To address the concern related to the lack of a physical examination, use of virtual stethoscopes, pulse oximeters, and home spirometry devices may complement a virtual visit (40). A program where patients can loan these devices to aid physical examinations may reduce socio-economic disparities in telehealth treatment outcomes among patients (41).

In qualitative responses, participants expressed a preference for virtual appointments without technological problems. Providers should seek to eliminate any technical issues on their end. Technological problems may be due to the slower Internet speed among youth of lower incomes. Disparities in access to and quality of telehealth exist (42–48). In the 2021 National Survey Trends in Telehealth Use in the United States, where 33.1% of participants were parents/caregivers, among telehealth users, the highest share of visits that used video took place among those earning at least $100,000 (68.8%) and non-Latino White individuals (61.9%). Video visits were lowest among those without a high school diploma, non-Latino Asian, and non-Latino Black individuals (42). In a study of pediatric telehealth in Chicago, incomplete video visit rates were associated with lower broadband access (43). To enhance satisfaction, increasing access to reliable Internet services and technological devices will be valuable. The Connected Care Pilot Program in the United States. is one example that seeks to cover expenses related to broadband connectivity and network equipment in selected pilot locations to decrease inequities in access to technology (49).

In a qualitative study of patients as young as 15 years, participants expressed appreciation for anonymity and privacy during telehealth appointments (23). Anonymity and privacy can be especially important to adolescents who are starting to have their own remote visits and spend time alone with the healthcare provider. In large households, anonymity and privacy can be problematic when participating in virtual appointments. Strategies to promote anonymity and privacy include medical providers asking adolescents to move to a private space or type questions and answers and asking others to leave the room (50). Independent telehealth use (without the presence of a parent) was conceptualized by referring to the “Conceptual framework of early adolescence” (51) as a strategy to help adolescents develop self-efficacy and thoughtful decision-making abilities (18).

### 4.5. Implications for future research

Much research focused on satisfaction with telehealth among parents and medical providers (34, 52–61). Research on satisfaction with telehealth among children and adolescents, especially per country, is very limited. Even younger patients as young as 5 (median age 5 to 10 years) can be interviewed through asking simple questions (20). Future research may assess reasons why young patients prefer certain characteristics of telehealth or in-person visits. Investigating differences in satisfaction among socio-demographic, cultural, and linguistic groups of children and adolescents is another needed area of study. Future studies may compare satisfaction among children and adolescents based on residence in rural and urban areas with a special emphasis on whether a fast/stable Internet connection is available. They may assess the effectiveness of telehealth compared to in-person services on satisfaction, quality of life, and health outcomes among youth. Parents expressed a preference of using video compared to in-person consultations for their children and adolescents due to the fear of contracting COVID-19 in past qualitative research during the pandemic (62). Future interviews with adolescents may ask regarding preferences for using remote compared to in-person care following the COVID-19 pandemic when fear of contracting a virus may not be as elevated. Based on the “Conceptual framework of early adolescence (18, 51),” an innovative area of research is to investigate how independent telehealth use affects adolescent’s self-efficacy.

### 4.6. Conclusion

Young people expressed high levels of satisfaction with telehealth while also preferring a combination of in-person and telehealth services. They realized the benefit of being in the same room with the medical provider. The systematic review offered recommendations for improving telehealth services specifically using video visits instead of telephone visits, enhancing technology connectivity quality, increasing availability of telehealth appointments, and ensuring anonymity and privacy for adolescents during telehealth appointments. Future RCTs, developed with the suggestions by children and adolescents, may improve satisfaction with telehealth and health outcomes.

## Author contributions

GDK conceived and designed the study. TC searched databases and removed duplicates. GDK, TC, SA, and BK reviewed abstracts and full-text articles. GDK, BK, and SA extracted data. GDK, BK, and SA performed risk of bias analysis. GDK wrote the first draft of the manuscript. GDK, TC, BK, and SA reviewed and modified manuscript. All authors contributed to the article and approved the submitted version.

## Funding

The GDK received the Frances Willson Thomson Fellowship by the University of Michigan-Flint to conduct research in the area of pediatric telehealth.

## Conflict of interest

The authors declare that the research was conducted in the absence of any commercial or financial relationships that could be construed as a potential conflict of interest.

## Publisher’s note

All claims expressed in this article are solely those of the authors and do not necessarily represent those of their affiliated organizations, or those of the publisher, the editors and the reviewers. Any product that may be evaluated in this article, or claim that may be made by its manufacturer, is not guaranteed or endorsed by the publisher.
