# Supplementary material for: Satisfaction with pediatric telehealth according to the opinions of children and adolescents during the COVID-19 pandemic: A literature review
Source: Front Public Health. 2023 Apr 6;11:1145486. doi: 10.3389/fpubh.2023.1145486 (PMC10118045; doi:10.3389/fpubh.2023.1145486)
Supplement: Supplementary file 1 [file Data_Sheet_1.docx]

**Additional file 1: Search strings by database^**

**PubMed**

**Steps: In** [**https://pubmed.ncbi.nlm.nih.gov/**](https://pubmed.ncbi.nlm.nih.gov/)**, type the entire search string into the Query**

**box under Advanced search**

((((("Pediatrics"[Mesh]) OR ("Child"[Mesh]) OR ("Adolescent"[Mesh]) OR (pediatric*[Title/Abstract]) OR (paediatric*[Title/Abstract]) OR (child*[Title/Abstract]) OR (teen*[Title/Abstract]) OR (adolescen*[Title/Abstract]))) AND ((("Telemedicine"[Mesh]) OR ("Remote Consultation"[Mesh]) OR (telemedicine[Title/Abstract]) OR ("video consultation"[Title/Abstract])OR ("remote consultation"[Title/Abstract]) OR (telehealth[Title/Abstract]) OR ("remote consultation"[Title/Abstract])))) AND ("COVID-19" OR "COVID-19"[MeSH Terms] OR "SARS-CoV-2" OR "sars-cov-2"[MeSH Terms] OR "Severe Acute Respiratory Syndrome Coronavirus 2" OR "NCOV" OR "2019 NCOV" OR ("coronavirus"[MeSH Terms] OR "coronavirus" OR "COV"))) AND ((((("Patient Satisfaction"[Mesh]) OR (satisfaction[Title/Abstract])) OR (attitude*[Title/Abstract])) OR (attitude*[Title/Abstract])) OR (perception*[Title/Abstract]))

**EMBASE**

**Steps: In the subscription-only database, place the entire search string in the box under**

**Advanced search and use all default settings.**

('pediatrics'/exp OR pediatrics OR 'child'/exp OR child OR 'adolescent'/exp OR adolescent OR pediatric*:ti,ab OR paediatric*:ti,ab OR child*:ti,ab OR teen*:ti,ab OR adolescen*:ti,ab) AND ('telemedicine'/exp OR telemedicine OR 'remote consultation'/exp OR 'remote consultation' OR telemedicine:ti,ab OR 'video consultation':ti,ab OR telehealth:ti,ab OR 'remote consultation':ti,ab) AND ('covid 19'/exp OR 'covid 19' OR 'sars cov 2'/exp OR 'sars cov 2' OR 'severe acute respiratory syndrome coronavirus 2'/exp OR 'severe acute respiratory syndrome coronavirus 2' OR ncov OR '2019 ncov'/exp OR '2019 ncov' OR 'coronavirus'/exp OR coronavirus OR cov) AND ('patient satisfaction'/exp OR 'patient satisfaction' OR satisfaction:ti,ab OR attitude*:ti,ab OR perception*:ti,ab)

**PsycINFO (Housed in EBSCO)**

**Steps: In the subscription-only database, under Advanced search, place each of the 4**

**strings in a separate search box, and use all default options.**

DE "Coronavirus" OR DE "COVID-19" OR TI ( "COVID-19" OR "SARS-CoV-2" OR "Severe Acute Respiratory Syndrome Coronavirus 2" OR "NCOV" OR "2019 NCOV" OR "coronavirus" OR "COV" ) OR AB ( "COVID-19" OR "SARS-CoV-2" OR "Severe Acute Respiratory Syndrome Coronavirus 2" OR "NCOV" OR "2019 NCOV" OR "coronavirus" OR "COV" )

AND

(DE "Pediatricians" OR DE "Pediatrics") OR (DE "Child Health") ) OR TI ( pediatric* OR paediatric* OR child* OR teen* OR adolescent* ) OR AB ( pediatric* OR paediatric* OR child* OR teen* OR adolescent* )

AND

(DE "Telemedicine" OR TI (telemedicine OR "remote consultation" OR "video consultation" OR telehealth ) OR AB ( telemedicine OR "remote consultation" OR "video consultation" OR telehealth )

AND

(DE "Satisfaction") OR (DE "Health Attitudes") ) OR TI ( satisfaction OR attitude* OR perception* ) OR AB ( satisfaction OR attitude* OR perception* )

Filter: exclude dissertations

**CINAHL (Housed in EBSCO)**

**Steps: In the subscription-only database, under Advanced search, place each of the 4**

**strings in a separate search box, and use all default options.**

((((((MH Pediatrics+)) OR ((MH Child+)) OR ((MH Adolescent+)) OR ((TI pediatric* OR AB pediatric*)) OR ((TI paediatric* OR AB paediatric*)) OR ((TI child* OR AB child*)) OR ((TI teen* OR AB teen*)) OR ((TI adolescen* OR AB adolescen*))))

AND

((((MH Telemedicine+)) OR ((MH "Remote Consultation+")) OR ((TI telemedicine OR AB telemedicine)) OR ((TI "video consultation" OR AB "video consultation")) OR ((TI "remote consultation" OR AB "remote consultation")) OR ((TI telehealth OR AB telehealth)) OR ((TI "remote consultation" OR AB "remote consultation")))))

AND

(COVID-19 OR (MH COVID-19+) OR SARS-CoV-2 OR (MH sars-cov-2+) OR "Severe Acute Respiratory Syndrome Coronavirus 2" OR NCOV OR "2019 NCOV" OR ((MH coronavirus+) OR coronavirus OR COV )))

AND

((((((MH "Patient Satisfaction+")) OR ((TI satisfaction OR AB satisfaction))) OR ((TI attitude* OR AB attitude*))) OR ((TI attitude* OR AB attitude*))) OR ((TI perception* OR AB perception*)))

^The PubMed database search string was translated so that the CINAHL and Embase databases search strings were comparable using the Polyglot Search in the Systematic Review Accelerator developed by the Institute for Evidence-Based Health Care (https://sr-accelerator.com/). The PsycINFO search string was translated so that it was comparable to the CINAHL search string using the Polyglot Search in the Systematic Review Accelerator.
