# Supplementary material for: Satisfaction with pediatric telehealth according to the opinions of children and adolescents during the COVID-19 pandemic: A literature review
Source: Front Public Health. 2023 Apr 6;11:1145486. doi: 10.3389/fpubh.2023.1145486 (PMC10118045; doi:10.3389/fpubh.2023.1145486)
Supplement: Supplementary file 2 [file Data_Sheet_2.docx]

**Additional file 2: Part 1. Flow chart indicating identification of studies via the PubMed, Embase, CINAHL, and PsycINFO databases**

**Identification of studies via databases**

Records removed *before screening*:

Duplicate records removed by automation tools

(n = 401)

Duplicate records removed by researchers: (n = 7)

Records marked as ineligible by automation tools (n = 5)

Records identified from:

PubMed (n = 381)

Embase (n = 686)

CINAHL (n = 101)

PsycINFO (n = 50)

Total (n = 1218)

**Identification**

Records excluded following title/abstract review

(n = 745)

Records screened

(n = 805)

Records sought for retrieval

(n = 60)

Records not retrieved

(n = 1)

**Screening**

Records excluded:

Wrong article or study type

(n = 23)

Full-text not in English (n = 1)

Not satisfaction or wrong topic (n = 12)

Focused on caregiver opinions only (n = 6)

Primary population of adults (n = 2)

Study conducted prior to the pandemic (n = 1)

Records assessed for eligibility

(n = 59)

Studies included in review

(n = 14)

**Included**

**Additional file 2: Part 2. Quality of evidence scores based on JBI critical assessment tool common questions in the 14 included studies**

|  | Were the criteria for inclusion in the sample clearly defined? | Were the socio-demographic characteristics of participants described in detail? | Was the time period (such as months and years) for telehealth services clearly defined? | Were satisfaction measures valid and reliable?^ | Were appropriate statistical/  qualitative analyses used? | Total score |
| --- | --- | --- | --- | --- | --- | --- |
| Allison et al., 2022^(18)^ | 1 | 1 (Various socio-demographic characteristics were available.) | 1 | 1 | 1 | 5 |
| Bassi et al., 2022^(19)^ | 1 | 0 (Information on age was available.) | 1 | 0.5 | 1 | 3.5 |
| Capri et al., 2021^(20)^ | 1 | 1 (Information on age, gender, socio-economic status, and parental education was available.) | 0 (Dates not specified: COVID-19 emergency) | 1 | 1 | 4 |
| Carroll et al., 2022^(21)^ | 1 | 0 (Information on age was available.) | 1 | 1 | 1 | 4 |
| Choi et al., 2022^(22)^ | 1 | 1 (Information on age, gender, area of residence, and current romantic relationship was available.) | 1 | 0 | 1 | 4 |
| Hawke et al., 2021^(23)^ | 1 | 1 (Information on age, gender, area of residence, first language spoken, born in Canada, and race/ethnicity was available.) | 1 | 1* | 1 | 5 |
| Mekori-Domachevsky et al., 2021^(24)^ | 1 | 0.5 (Information on age and gender was available.) | 1 | 1 | 1 | 4.5 |
| Mikesell et al., 2022^(25)^ | 1 | 1 (Various socio-demographic characteristics were available.) | 1 | 1 | 1 | 5 |
| Randall et al., 2022^(26)^ | 1 | 0.5 (Information on age and gender was available.) | 1 | 0 | 1 | 3.5 |
| Spigel et al., 2021^(27)^ | 1 | 1 (Information on age, gender, and race/ethnicity was available.) | 1 | 0 | 1 | 4 |
| Stewart et al., 2021^(28)^ | 1 | 0 (Information on age was available.) | 1 | 0 | 1 | 3 |
| Troncone et al., (2022)^(29)^ | 1 | 0.5 (Information on age and gender was available.) | 1 | 1 | 1 | 4.5 |
| Wasilewska et al., 2022^(30)^ | 1 | 0.5 (Information on age and gender was available.) | 1 | 0 | 1 | 3.5 |
| Ziani et al., 2022^(31)^ | 1 | 0 (Information on age was available.) | 1 | 1* | 1 | 4 |

^A score of 0 indicated that validity and/or reliability were not discussed. A score of 0.5 meant that the authors modified the survey from existing surveys with some reliability or validity data. A score of 1 meant that at least some measure of reliability or validity was presented.

*Not applicable since interviews were used.
